# Variations and characteristics of quality indicators for maintenance hemodialysis patients: A systematic review

**DOI:** 10.1002/hsr2.89

**Published:** 2018-09-05

**Authors:** Kakuya Niihata, Sayaka Shimizu, Yasushi Tsujimoto, Tatsuyoshi Ikenoue, Shunichi Fukuhara, Shingo Fukuma

**Affiliations:** ^1^ Department of Hygiene and Preventive Medicine School of Medicine, Fukushima Medical University Fukushima Japan; ^2^ Center for Innovative Research for Communities and Clinical Excellence (CiRC^2^LE) Fukushima Medical University Fukushima Japan; ^3^ Department of Healthcare Epidemiology, School of Public Health in the Graduate School of Medicine Kyoto University Kyoto Japan; ^4^ Department of Nephrology and Dialysis, Kyoritsu Hospital Hyogo Japan; ^5^ Human Health Sciences, Graduate School of Medicine Kyoto University Kyoto Japan

**Keywords:** clinical nephrology, epidemiology, quality improvement

## Abstract

**Aims:**

Several quality indicators (QIs) to improve the quality of practice for hemodialysis patients have been implemented. However, the variations and characteristics of these indicators in terms of their use and feasibility have not been investigated. We conducted a systematic review to evaluate the variations and characteristics of existing QIs for maintenance hemodialysis patients.

**Methods:**

We conducted a systematic literature search of MEDLINE via PubMed, Scopus, the Cochrane Library, and CINAHL, without date limits, on February 26, 2016. We selected the English‐written articles regarding QIs for patients aged ≥18 years who were on maintenance hemodialysis therapy ≥3 months, and extracted the definition and development process of the reported QIs. We categorized each indicator into one of four types, namely, structure, process, surrogate outcome, and outcome, and assessed the data sources that were necessary to measure it.

**Results:**

We included 70 articles and identified 101 indicators, and found that most of the consensus processes for selecting indicators were unclear. We also found that most indicators were not process indicators and that the measurement of some indicators required a chart review, which limits their use and feasibility.

**Conclusions:**

Development of QIs for hemodialysis patients in the future should use a definitive consensus process and consider process‐centered indicators that can be measured automatically using claims data and test results contained in electronic medical records, to improve usability and feasibility.

## 1 INTRODUCTION

Treatment of patients receiving maintenance hemodialysis is subject to large variations in practice patterns.^1^, ^2^, ^3^, ^4^, ^5^ Given that such variation is associated with the clinical outcomes of these patients,^4^, ^6^, ^7^ improving the quality of practice is critical to their management. To achieve this, quality indicators (QIs) are used as precise measures of quality. These have been used for patients with various diseases, including end‐stage renal disease (ESRD).^8^, ^9^ However, while several studies have reported associations between the use of effective QIs and clinical outcomes in hemodialysis patients,^10^, ^11^, ^12^ the methods by which these QIs were established in these studies are not clear, and we cannot, therefore, be sure that their selection was based on scientifically valid methods.

In setting QIs, many have recognized the usefulness of Donabedian's framework, which defines quality measurement of health care in three parts: structure, process, and outcome.^13^ This framework is sometimes expanded into four parts to include a surrogate outcome.^14^ As each part is associated with its own advantages and disadvantages, quality can be precisely measured if the meaning of each part differs according to the aim of the measurement initiative.^14^ In addition to variations in types of QIs, there are also differences in the process of developing QIs, for example, in the use of guideline‐based versus Delphi methods. The Delphi method was originally developed to ensure an anonymous consensus to avoid domination by a few experts; however, even this method has some variations.^15^ Moreover, although there are many variations in the components of QIs and in their development process, no systematic review of existing QIs for maintenance hemodialysis patients has yet been conducted, unlike the case of other areas such as palliative care,^16^ trauma care,^17^ and anesthesia.^18^

Here, we conducted a systematic review of QIs for maintenance hemodialysis patients to construct item lists and to identify the pros and cons of existing QIs. Our findings will help improve the future development of QIs.

## 2 MATERIALS AND METHODS

This current systematic review was conducted according to the Preferred Reporting Items for Systematic Reviews and Meta‐Analyses (PRISMA) statement.^19^ The study protocol was not registered in the PROSPERO because some standard methods of the systematic review process (eg, prespecification of the primary outcomes, risk of bias assessment, data synthesis including meta‐analyses, or evidence synthesis using GRADE approach), which should be stated through the registration process in PROSPERO, were not required in this study.

### 2.1 Literature search

We conducted a systematic literature search of MEDLINE via PubMed, Scopus, the Cochrane Library, and CINAHL, without date limits, on February 26, 2016. Our search strategy is shown in Text S1. We checked the references of all potential publications to extract the definition and development process of the reported QIs.

### 2.2 Study selection

We included and excluded publications according to the following criteria:

1. Only English‐written publications were included.
2. Those that described the development process or characteristics of QIs for patients aged ≥18 years who were on maintenance hemodialysis therapy ≥3 months were included. Publications examining patients using special modalities such as nocturnal hemodialysis, home dialysis, and combination therapy with peritoneal dialysis were excluded.
3. Those describing only the QIs that should be achieved on initiation of hemodialysis, such as arteriovenous fistula (AVF) creation during the initiation of hemodialysis, were excluded because these QIs could not be modified during the maintenance hemodialysis phase. These QIs were also excluded from the extracting items for each QI set, which we defined as a set of QIs examined in each included article.
4. Those not describing the rationale behind associations with the QIs were excluded. Those that discussed the rationale, such as that behind the association between the QI and clinical outcomes, but did not cite a reference(s), were included.
5. Those in which the numerators and denominators of the QIs were defined, or could be deduced from the description of the QIs, were included.
6. Those that described indicators with specific goals were included. For example, the target hemoglobin (Hb) level, such as Hb ≥ 10 g/dL, had to be reported when Hb level was a QI.
7. Those describing QIs for primary care settings were excluded.
8. Editorials, letters, comments, case reports, dissertations, and theses were excluded.

Four authors (I.T., S.S., N.K., and T.Y.) were divided into two teams, with I.T. and N.K. in one, and S.S. and Y.T. in the other. Each team examined half of the articles identified by the electronic search strategy described above and checked them according to the inclusion/exclusion criteria. The two members of each team reviewed each article independently. Articles that were considered to meet the inclusion criteria were obtained as full articles and independently reassessed for inclusion as described above. In the case of discordance in the selection of an article within one team, one author from the other team assessed its inclusion.

### 2.3 Data extraction

We used a structured Excel data collection form designed by the authors to independently extract the required data from the included studies. Extracted data included the consensus process used to develop the indicator, references for the indicator, a general description of the items in each QI set, the types of indicators for each item, the data sources used to measure each item, and the clinical practice guidelines supporting each QI. We categorized each indicator into one of four types: structure, process, surrogate outcome, and outcome. We defined structure indicators as hospital or clinical resources such as the number of doctors and nurses. We defined process indicators as those that can only be modified by health care professionals and do not depend on the patient's condition, such as the frequency of blood tests and noninvasive procedures. We defined surrogate outcomes and outcomes as patient conditions, with surrogate outcomes represented by clinical signs such as test results that are associated with outcomes. We also categorized each indicator according to the data sources that were necessary to measure that indicator, such as claims data, test results, and medical chart review. We defined claims data as data such as information on a disease, procedure, or prescription.

As this is a systematic review of the literature, approval by the research ethics committee was not required.

## 3 RESULTS

### 3.1 Study selection

**Figure 1:**
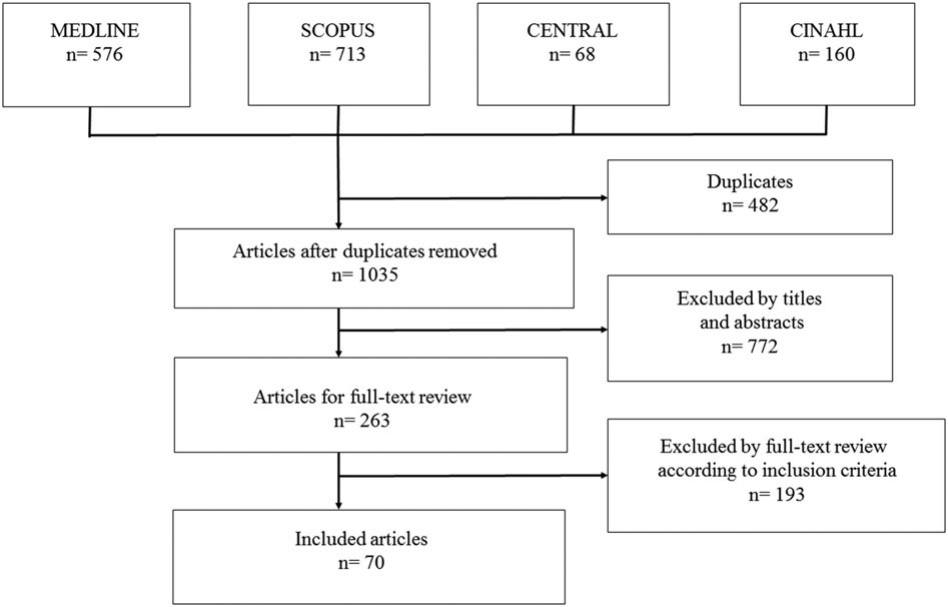
Flowchart of study selection

Following the removal of duplicate publications (576 from MEDLINE, 713 from Scopus, 68 from the Cochrane Library, and 160 from CINAHL), 1,035 articles were retrieved from the electronic literature search. Two hundred and sixty‐three full‐text articles were selected after title‐abstract review and assessed for eligibility according to the inclusion criteria. Seventy articles were included for data extraction after the following articles were excluded: 7 articles written in languages other than English, 134 articles of unsuitable publication type, 13 articles containing unsuitable target populations, 11 articles that did not use QIs, 19 articles with a lack of rationale, 5 articles with vague denominators and nominators, and 4 articles for not setting specific goals for QIs. The flowchart of the study selection process is shown in Figure 1, and the references of the included articles are shown in Text S2. Some QI sets were addressed in several articles. Among the 70 articles selected, 30 used indicators developed by the Centers of Medicare and Medicaid Services' “End‐Stage Renal Disease (ESRD) Core Indicator Project” and “Clinical Performance Measures (CPM) project,” which have now been merged into the CPM project in the United States. Details of the CPM project were described on its website,^20^ from which we extracted the associated items. Quality indicator sets implemented by some health care providers, such as Fresenius Medical Care and Davita, were addressed in several articles.^21^, ^22^ The details of QIs other than those for CPM regarding their developer, consensus process, and references associated with their development are summarized in Table 1.

**Table 1:** Development process of quality indicators except for clinical performance measures

Article	Organization	Consensus process	Reference
Patton S et al (1)	St. Michael Hospital, US	Unknown	Standards of care or practice guideline
Armistead N et al (2)	Mid‐Atlantic renal coalition, US	Unknown	Unknown
Bogdanski P et al (3)	St Joseph's Health Care, UK	Meeting of the interdisciplinary task force at St Joseph's Health Care	Unknown
Bonucchi D et al (4)	University Hospital, Modena, Italy	Unknown	Previous studies
Capelli JP (5)	Our Lady of Lourdes Medical Center, US	Meeting of the quality assessment team at our lady of Lourdes medical center	HCFA case mix indicators
Coelho AP et al (6)	National Commission for Monitoring of Dialysis, Portugal	Unknown	KDOQI
Cormier T et al (7)	Southern Alberta Renal Program, Canada	Unknown	KDOQI
Diamant MJ et al (8)	Authors	Unknown	Previous studies
Grangé S et al (9)	Authors	Unknown	KDOQI, EBPG
Hirth RA et al (10)	Authors	Unknown	KDOQI, CPM
Kõlvald K et al (11)	Authors	Unknown	EBPG, KDIGO, local guideline
Lacson E et al (12)	Fresenius Medical Care North America	Unknown	National guideline
Lowrie EG (13)	Authors	Statistical model: The indicators associated with 1‐year mortality were selected.	Previous studies
Lynch SK et al (14)	Authors	Unknown	Unknown
Morsch CM et al (15)	Authors	Unknown	KDOQI, previous studies
Mozes B et al (16)	Authors	Statistical model: The indicators associated with 1‐year mortality were selected.	Previous studies
Parra E et al (17)	Authors	Meeting of four hemodialysis volunteer Spanish centers	Unknown
Peter J et al (18)	Milford Dialysis Unit, US	Unknown	KDOQI
Plantinga LC et al (19)	Authors	Unknown	KDOQI
Plantinga LC et al (20)	Authors	Unknown	KDOQI, CSN guideline, EBPG
Richards N et al (21)	Fresenius Medical Care	Unknown	EBPG
Saudan P et al (22)	Authors	Unknown	Unknown
Tan J (23)	Authors	Unknown	Unknown
Thompson S et al (24)	Authors	Unknown	KDOQI, CSN guideline
Wazny LD et al (25)	Authors	Unknown	KDOQI, CSN guideline
Wilson SM et al (26)	Authors	Unknown	Unknown
Wintz R et al (27)	PQRI Kidney Associates, US	Unknown	Unknown
Benner D et al (28)	DaVita, US	Unknown	Previous studies
Couchoud C et al (29)	QUEST	Unknown	EBPG
Di Benedetto A et al (30)	Authors	Unknown	EBPG
Hoar S et al (31)	Authors	Unknown	Previous studies
Ilumin MP et al (32)	Primary Nurse Monthly Summary, Canada	Unknown	KDOQI
Lindberg M et al (33)	Authors	Unknown	Previous studies
Ludvigsen MS et al (34)	Authors	Unknown	Previous studies
Marcelli D et al (35)	Authors	Unknown	Unknown
Ponce P et al (36)	NephroCare, Portugal	Unknown	International guidelines
Saudan P et al (37)	Authors	Unknown	Unknown
Van Andringa de Kempenaer T et al (38)	Rijnland Hospital, the Netherlands	Unknown	KDOQI, previous studies
Waeleghem JP et al (39)	ORPADT, the professional Nephrology Nurses Association of Flanders, Belgium	Unknown	Unknown
Yuan CM et al (40)	Authors	Unknown	Unknown

Abbreviations: CPM, clinical performance measure; KDOQI, kidney disease outcomes quality initiative; EBPG, European best practice guideline; CSN, Canadian society of nephrology; QUEST, quality European studies; ORPADT, the organization of paramedical personnel of the dialysis and transplantation centers. The reference article numbers refer to the list of included articles shown in Text S2.

### 3.2 Variations in the development of QI sets

Most QI sets were developed by experts' consensus based on international guidelines such as the National Kidney Foundation‐Dialysis Outcomes Quality Initiative (NKF‐DOQI) Clinical Practice Guidelines^23^ and the European Best Practice Guidelines (EBPG).^24^ In terms of the CPM project, the first 16 QIs were developed based on the NKF‐DOQI Clinical Practice Guidelines. These indicators have been updated in recent expert meetings, and the process is disclosed on their website.^20^ Compared to QIs developed according to the CPM project, the selection process for most other QI sets that were developed according to the relevant guidelines and the consensus process of experts' meetings is unclear, because they have not been published or disclosed in English‐language articles. Although the consensus processes for QIs for CPM have been disclosed, these were qualitative rather than quantitative processes using methods like the Delphi method.

### 3.3 Characteristics of QI items

One hundred one QI items were identified among the included articles after identical indicators with different ideal values had been combined. Characteristics of the QI items referenced by more than one article are summarized in Table 2. The detailed characteristics of all QI items are shown in Table S1. The clinical practice guidelines or performance measures supporting the indicators, such as CPM,^20^ EBPG,^24^ and the clinical practice guideline endorsed by Japanese society of dialysis therapy,^25^ are also shown in Table 2. These items were categorized into 10 areas: anemia, mineral and bone disorder (MBD), dialysis adequacy, vascular access, nutrition, fluid management, diabetes, dyslipidemia, infection, and others. The QIs for anemia, MBD, dialysis adequacy, vascular access, nutrition, and fluid management have been examined in several studies (Table S1). Most of these indicators were for surrogate outcomes, such as achievement of hemoglobin level, serum calcium level and Kt/V, and maximizing the use of AVF, whereas CPM indicators were predominantly process indicators, such as assessment of iron status, and measurement of serum calcium level and hemodialysis adequacy. In particular, although QIs for nutrition were among the most frequently used, they only assessed surrogate outcomes.

**Table 2:** Characteristics of included quality indicators

Item	Category	Data Source	Referenced CPGs and Performance Measures
Anemia			
Achievement of Hb (or Ht) level	Surrogate outcome	Blood test	CPM, JSDT, EBPG
Achievement of Hb (or Ht) level on ESA therapy	Surrogate outcome and claims data	Blood test	CPM, JSDT, EBPG
Achievement of ferritin level	Surrogate outcome	Blood test	JSDT, EBPG
Achievement of TSAT	Surrogate outcome	Blood test	JSDT, EBPG
Assessment of iron status	Process	Claims data or blood test	CPM, JSDT, EBPG
Use of iron therapy when indicated	Process	Claims data and blood test	CPM, EBPG
Use of iron therapy in iron overload	Process	Claims data and blood test	CPM, EBPG
Administration of ESA	Process	Claims data	CPM, EBPG
Mineral bone disorder			
Achievement of Ca level	Surrogate outcome	Blood test	CPM, JSDT, EBPG
Achievement of P level	Surrogate outcome	Blood test	CPM, JSDT, EBPG
Achievement of Ca and P product	Surrogate outcome	Blood test	CPM,
Achievement of PTH level	Surrogate outcome	Blood test	JSDT, EBPG
Achievement of ALP level	Surrogate outcome	Blood test	
Measurement of Ca level	Process	Claims data or blood test	CPM, JSDT
Measurement of P level	Process	Claims data or blood test	CPM, JSDT
Dialysis adequacy			
Achievement of Kt/V	Surrogate outcome	Chart review	CPM, JSDT, EBPG
Achievement of URR	Surrogate outcome	Blood test	CPM,
Achievement of Kt	Surrogate outcome	Chart review	
Measurement of adequacy	Process	Claims data or blood test	CPM, JSDT, EBPG
Method of measurement of delivered dose	Process	Chart review	CPM
Dialysis time	Surrogate outcome	Chart review	JSDT, EBPG
Number of dialysis sessions	Process	Claims data	
Vascular access			
Maximizing use of AVF	Surrogate outcome	Chart review	CPM, JSDT, EBPG
Minimizing use of catheter	Surrogate outcome	Chart review	CPM, EBPG
Functional autogenous AVF or referral to vascular surgeon for placement	Surrogate outcome	Chart review	CPM
Catheter vascular access and referred for vascular evaluation for permanent access	Surrogate outcome	Chart review	CPM
Decision‐making by surgeon to maximize placement of autogenous AVF	Process	Chart review	CPM
Nutrition			
Achievement of albumin level	Surrogate outcome	Blood test	CPM, EBPG
Fluid management			
Blood pressure control	Surrogate outcome	Chart review	JSDT
Intradialytic hypotension	Surrogate outcome	Chart review	JSDT, EBPG
Change in body weight between dialysis sessions	Surrogate outcome	Chart review	JSDT
Ultrafiltration rate	Surrogate outcome	Chart review	JSDT
Dietary sodium reduction advice	Process	Chart review	CPM
Sodium profiling practice for hemodialysis	Process	Chart review	CPM
Restriction of dialysate sodium	Process	Chart review	CPM
Periodic assessment of postdialysis weight by nephrologists	Process	Chart review	CPM
Diabetes			
Measurement of blood sugar status	Process	Claims data	CPM
Dyslipidemia			
Achievement of cholesterol level	Surrogate outcome	Blood test	JSDT
Measurement of lipid status	Process	Claims data	CPM
Infection			
Influenza immunization	Process	Chart review	CPM
Suspected infection	Surrogate outcome	Chart review	CPM
Clinically established infection	Surrogate outcome	Chart review	CPM
Hemodialysis vascular access‐related infection	Surrogate outcome	Chart review	CPM,
Hemodialysis vascular access‐related bacteremia	Surrogate outcome	Chart review	CPM,
Hemodialysis catheter‐related infection	Surrogate outcome	Chart review	CPM
Hemodialysis catheter‐related bacteremia	Surrogate outcome	Chart review	CPM
Hemodialysis arteriovenous graft‐related infection	Surrogate outcome	Chart review	CPM
Hemodialysis AVF‐related infection	Surrogate outcome	Chart review	CPM
Clinically established infections resulting in hospitalization	Outcome	Chart review	CPM
Hemodialysis vascular access‐related infections resulting in hospitalization	Outcome	Chart review	CPM
Hemodialysis catheter‐related infections resulting in hospitalization	Outcome	Chart review	CPM
Others			
Mortality	Outcome	Chart review	
Hospital admission	Outcome	Claim data	
Achievement of potassium level	Surrogate outcome	Blood test	
Achievement of bicarbonate level	Surrogate outcome	Blood test	
Water quality test	Process	Chart review	
Attestation of patient satisfaction survey	Process	Chart review	CPM
CAHPS in‐center‐hemodialysis survey	Process	Chart review	CPM
Assessment of health‐related quality of life	Process	Chart review	CPM

Abbreviations: CPGs, clinical practice guidelines; Hb, hemoglobin; Ht, hematocrit; CPM, clinical performance measure; JSDT, Japanese society of dialysis therapy; EBPG, European best practice guideline; ESA, erythropoietin stimulating agent; TSAT, transferrin saturation; Ca, calcium; P, phosphorus; PTH, parathyroid hormone; ALP, alkaline phosphatase; URR, urea reduction ratio; AVF, arteriovenous fistula; HbA1c, hemoglobin A1c; CRP, C‐reactive protein; CHAPS, consumer assessment of health care providers and systems; HCFA, Health Care Finance Administration. The indicators referenced as CPM include those related to Health Care Finance Administration or Centers for Medicare and Medicaid Services.

### 3.4 Variations in data resources

The data sources used to measure each QI are summarized in Tables 2 and S1. Quality indicators for anemia and MBD can all be measured using information in claims data or blood tests. In contrast, several QIs for other areas need measurement using a chart review. For example, measurement of Kt/V or ultrafiltration rate requires detailed information such as dialysis time, postdialysis body weight, and ultrafiltrate volume, which can rarely be retrieved from test results or claims data. Furthermore, a chart review is needed to measure QIs for infection due to the need for diagnostic information.

## 4 DISCUSSION

We conducted a systematic review and generated an items list of existing QIs for adult maintenance hemodialysis patients to determine the pros and cons associated with their use and to discuss the requirements for the development of future QIs. We evaluated variations in the areas and types of indicators and the associated development processes. We also categorized the source data to measure each item in the QI sets. From the perspective of this information, we then assessed the pros and cons of the existing QIs for maintenance hemodialysis patients.

Most QI sets fell under the following areas: anemia, MBD, dialysis adequacy, vascular access, nutrition, fluid management, and infection. Most of the QIs for these areas measured surrogate outcomes, and in particular, indicators for nutrition comprised only surrogate outcomes. Although outcome indicators are, generally, intuitive and easy to understand, in practice, they often require long‐term observation to detect changes. Furthermore, they often require case‐mix adjustment, because they are easily influenced by a patient's condition.^14^ In contrast, process indicators are so useful for detecting changes in practice within a short period of time, that those most associated with relevant outcomes have been recommended for the assessment of quality of care.^8^, ^14^ Additionally, the inclusion of more process indicators may benefit maintenance hemodialysis patients. In particular, as our findings show that indicators of nutrition include only surrogate outcomes, process indicators such as nutritional support should be included.

We found that there were several variations in the development process of QIs for maintenance hemodialysis patients. Most of the QI sets were developed by expert consensus or author definition based on international guidelines. Although there is no gold standard guideline‐based method,^26^ the development processes used by the studies included in this review were obscure. Most indicators developed using expert consensus were developed by specific health care providers, and the detailed consensus process was not made publicly available. Of the indicators developed using expert consensus, the development process of CPM developed by Medicare were disclosed in a peer‐reviewed article^27^ and on their website.^20^ The consensus was achieved by face‐to‐face discussion, and the evaluation was descriptive rather than using a quantitative approach such as the Delphi method. A previous report suggested that a consensus achieved using only a face‐to‐face discussion could be biased toward the opinions of dominant persons or groups, owing to the difficulty in assuring anonymity in such processes.^15^ To develop a validated set of QIs, it may be important to clarify the consensus process and to exclude such dominance.

The feasibility of measure indicators may be important in the selection of QI sets. Health care providers reportedly spend large sums of money to report their QIs.^8^ Several indicators for maintenance hemodialysis patients extracted in this review, even in pivotal areas such as hemodialysis adequacy and fluid management, require a chart review for measurement. These QIs, therefore, require human resources for measurement, which may place a substantial burden on health care providers. Quality indicator sets for maintenance hemodialysis patients that do not require a chart review are warranted. In recent years, most blood test and claims data have been managed in a database, which allows for the automatic retrieval of information required to measure QIs. Moreover, several types of dialysis management software have been developed to electronically manage individuals' data during dialysis sessions.^28^ The use of this software and data can improve current QI measures that require a chart review.

The present systematic review has several strengths with respect to its impact on the health care of hemodialysis patients and methodology. First, it is the first systematic review to examine QIs for maintenance hemodialysis patients. End‐stage renal disease is a leading area in which QIs have been used for clinical practice and insurance systems.^29^ No systematic review of QIs for maintenance hemodialysis patients has been conducted, and the QI sets and their items, as well as the development processes used, have not been reported. Second, the four authors were divided into two teams, which reviewed the articles identified through a systematic literature search. Each member of the two teams reviewed half of the articles, and in the case of disagreement, a member of the other team reviewed the inclusion, which mitigated any potential bias that may have resulted from dominance by one author in the consensus process.

This systematic review also had several minor limitations. First, we included only English‐language publications. Although non–English‐speaking countries have also developed indicators, English‐speaking countries such as the United States, Australia, Canada, and the United Kingdom have led the development of QIs,^18^ which suggests that the number of indicators we missed might be small. Second, we excluded QIs used for primary‐care settings. As patients with nondialysis chronic kidney disease (CKD) are often managed in primary care settings, QIs for the nondialysis CKD care phase, such as AVF creation, could have been included for primary‐care settings. However, as we focused on QIs that could be modified with maintenance hemodialysis in the ESRD care phase, and maintenance hemodialysis—which requires special equipment and registered medical specialists—can rarely be conducted in primary‐care settings, we considered that QIs for these settings may not be relevant. Finally, we conducted the systemic literature search without using EMBASE. Although medical subject headings cannot be used in Scopus, it covers most of the literatures in EMBASE,^30^ which assures that we minimally missed the indicators from EMBASE. Furthermore, our literature search covered the indicators in nursing practices by conducting the search via CINAHL.

In conclusion, this systematic review provides a detailed overview of the existing QIs for maintenance hemodialysis patients. To date, QIs for various areas have been developed and used for maintenance hemodialysis patients. While these indicators cover important factors associated with maintenance hemodialysis patients, most are surrogate outcome or outcome indicators. In contrast, process indicators, which detect changes in practice to measure quality of care, are sparse. Furthermore, the development processes have rarely been disclosed in detail and some indicators require a chart review for measurement, which limits their use and feasibility. Future development of QIs for maintenance hemodialysis patients should use definitive consensus processes and consider process‐centered indicators, which can be measured automatically using claims data and test results contained in electronic medical records, to improve usability and feasibility.

## FUNDING

This work was supported by “SPIRITS” (Supporting Program for Interaction‐based Initiative Team Studies) 2018 of Kyoto University and JPSS KAKENHI grant 23390130.

## CONFLICTS OF INTEREST

All authors have no conflicts of interest.

## AUTHOR CONTRIBUTIONS

Conceptualization: Kakuya Niihata

Investigation: Kakuya Niihata, Sayaka Shimizu, Yasushi Tsujimoto, Tatsuyoshi Ikenoue

Formal analysis: Kakuya Niihata, Sayaka Shimizu

Funding acquisition: Shingo Fukuma, Shunichi Fukuhara

Supervision: Shingo Fukuma

Writing—original draft: Kakuya Niihata

Writing—review and editing: Sayaka Shimizu, Yasushi Tsujimoto, Tatsuyoshi Ikenoue, Shingo Fukuma, Shunichi Fukuhara

## Supporting information

Table S1. Detailed characteristics of included quality indicators

Text S1. Search strategies for electronic literature search

Text S2. References of the included articles
