# Supplementary material for: Variations and characteristics of quality indicators for maintenance hemodialysis patients: A systematic review
Source: Health Sci Rep. 2018 Sep 5;1(11):e89. doi: 10.1002/hsr2.89 (PMC6242363; doi:10.1002/hsr2.89)
Supplement: Supplementary file 1 — Table S1. Detailed characteristics of included quality indicators [file HSR2-1-e89-s001.docx]

Supplementary Table 1. Detailed characteristics of included quality indicators

| Item | Category | Data source | Reference |
| --- | --- | --- | --- |
| Anemia |  |  |  |
| Achievement of Hb (or Ht) level | Surrogate outcome | Blood test | CPM, 2, 3, 6, 7, 8, 9, 10, 12, 13, 15, 17, 18, 19, 20, 21, 22, 24, 26, 27, 28, 30, 32, 36, 37, 39 |
| Achievement of Hb (or Ht) level on ESA therapy | Surrogate outcome and claims data | Blood test | CPM |
| Achievement of ferritin level | Surrogate outcome | Blood test | 3, 6, 7, 17, 18, 24, 32, 36 |
| Achievement of TSAT | Surrogate outcome | Blood test | 3, 7, 18, 24, 32 |
| Assessment of iron status | Process | Claims data or blood test | CPM |
| Use of iron therapy when indicated | Process | Claims data and blood test | CPM |
| Use of iron therapy in iron overload | Process | Claims data and blood test | CPM |
| Administration of ESA | Process | Claims data | CPM |
| Mineral bone disorder |  |  |  |
| Achievement of Ca level | Surrogate outcome | Blood test | CPM, 5, 7, 9, 17, 24, 25 |
| Achievement of P level | Surrogate outcome | Blood test | CPM, 5, 6, 7, 9, 11, 12, 17, 21, 25, 28, 36 |
| Achievement of Ca and P product | Surrogate outcome | Blood test | CPM, 8, 17, 19, 20, 21, 22, 31, 37 |
| Achievement of PTH level | Surrogate outcome | Blood test | 3, 7, 9, 11, 25, 28 |
| Achievement of ALP level | Surrogate outcome | Blood test | 3, 7 |
| Measurement of Ca level | Process | Claims data or blood test | CPM |
| Measurement of P level | Process | Claims data or blood test | CPM |
| Achievement of aluminum level | Surrogate outcome | Blood test | 3 |
| Achievement of vitamin D level | Surrogate outcome | Blood test | 9 |
| Dialysis adequacy |  |  |  |
| Achievement of Kt/V | Surrogate outcome | Chart review | CPM, 6, 7, 8, 9, 11, 12, 15, 17, 19, 20, 21, 22, 24, 26, 28, 36, 37, 39 |
| Achievement of URR | Surrogate outcome | Blood test | CPM, 2, 5, 7, 10, 11, 13, 23, 27, 32 |
| Achievement of Kt | Surrogate outcome | Chart review | 9, 13 |
| Target of creatinine reduction | Surrogate outcome | Blood test | 5 |
| Measurement of adequacy | Process | Claims data or blood test | CPM |
| Method of measurement of delivered dose | Process | Chart review | CPM |
| Dialysis time | Surrogate outcome | Chart review | 6, 21, 29, 36 |
| Number of dialysis sessions | Process | Claims data | 6, 21, 36 |
| Achievement of β_2_ microglobulin | Surrogate outcome | Blood test | 9 |
| Infusion volume | Process | Chart review | 9 |
| Use of high-flux membrane | Process | Chart review | 21 |
| Vascular access |  |  |  |
| Maximizing use of AVF | Surrogate outcome | Chart review | CPM, 2, 8, 13, 17, 19, 20, 22, 23, 37, 38 |
| Minimizing use of catheter | Surrogate outcome | Chart review | CPM, 2, 4, 9, 12, 13, 26, 28, 37, 38 |
| Functional autogenous AVF or referral to vascular surgeon for placement | Surrogate outcome | Chart review | CPM |
| Catheter vascular access and referred for vascular evaluation for permanent access | Surrogate outcome | Chart review | CPM |
| Decision-making by surgeon to maximize placement of autogenous AVF | Process | Chart review | CPM |
| Access flow rate | Surrogate outcome | Chart review | 7 |
| Compliance with 1^st^ appointment among patients with AVF creation | Surrogate outcome | Claims data | 14 |
| Compliance with 2^nd^ appointment among patients with AVF creation | Surrogate outcome | Claims data | 14 |
| Compliance with fistulogram among patients with AVF creation | Surrogate outcome | Claims data | 14 |
| Preoperative dialysis duplex ultrasound imaging | Surrogate outcome | Claims data | 14 |
| Nutrition |  |  |  |
| Achievement of albumin level | Surrogate outcome | Blood test | CPM, 5, 6, 7, 8, 9, 11, 12, 13, 15, 17, 19, 20, 22, 26, 28, 32, 36, 37 |
| Loss of body weight | Surrogate outcome | Chart review | 5 |
| Body mass index | Surrogate outcome | Chart review | 11 |
| Weight/Height | Surrogate outcome | Chart review | 13 |
| Fluid management |  |  |  |
| Blood pressure control | Surrogate outcome | Chart review | 7, 9, 13, 22, 37 |
| Intradialytic hypotension | Surrogate outcome | Chart review | 3, 9 |
| Change in body weight between dialysis sessions | Surrogate outcome | Chart review | 7, 33 |
| Ultrafiltration rate | Surrogate outcome | Chart review | 33, 34 |
| Target weight achievement | Surrogate outcome | Chart review | 7 |
| Dietary sodium reduction advice | Process | Chart review | CPM |
| Sodium profiling practice for hemodialysis | Process | Chart review | CPM |
| Restriction of dialysate sodium | Process | Chart review | CPM |
| Periodic assessment of post-dialysis weight by nephrologists | Process | Chart review | CPM |
| Diabetes |  |  |  |
| Achievement of HbA1c level | Surrogate outcome | Blood test | 7 |
| Measurement of blood sugar status | Process | Claims data | CPM, 7 |
| Dyslipidemia |  |  |  |
| Achievement of cholesterol level | Surrogate outcome | Blood test | 7, 9, 11 |
| Measurement of lipid status | Process | Claims data | CPM |
| Infection |  |  |  |
| Influenza immunization | Process | Chart review | CPM |
| Suspected infection | Surrogate outcome | Chart review | CPM |
| Clinically established infection | Surrogate outcome | Chart review | CPM |
| Hemodialysis vascular access-related infection | Surrogate outcome | Chart review | CPM, 3 |
| Hemodialysis vascular access-related bacteremia | Surrogate outcome | Chart review | CPM, 3 |
| Hemodialysis catheter-related infection | Surrogate outcome | Chart review | CPM |
| Hemodialysis catheter-related bacteremia | Surrogate outcome | Chart review | CPM |
| Hemodialysis arteriovenous graft-related infection | Surrogate outcome | Chart review | CPM |
| Hemodialysis AVF-related infection | Surrogate outcome | Chart review | CPM |
| Clinically established infections resulting in hospitalization | Outcome | Chart review | CPM |
| Hemodialysis vascular access-related infections resulting in hospitalization | Outcome | Chart review | CPM |
| Hemodialysis catheter-related infections resulting in hospitalization | Outcome | Chart review | CPM |
| Staff who had received at least 3 doses of the hepatitis B vaccine | Process | Chart review | 2 |
| Others |  |  |  |
| Mortality | Outcome | Chart review | 6, 16, 26, 28, 36 |
| Hospital admission | Outcome | Claim data | 6, 36 |
| Achievement of potassium level | Surrogate outcome | Blood test | 5, 7, 13 |
| Achievement of bicarbonate level | Surrogate outcome | Blood test | 9, 13 |
| Water quality test | Process | Chart review | 6, 36 |
| Achievement of CRP level | Surrogate outcome | Blood test | 11 |
| Achievement of lymphocyte count | Surrogate outcome | Blood test | 13 |
| Achievement of neutrophil count | Surrogate outcome | Blood test | 13 |
| Attestation of patient satisfaction survey | Process | Chart review | CPM |
| Achievement of patient satisfaction | Surrogate outcome | Chart review | 39 |
| CAHPS In-Center-Hemodialysis Survey | Process | Chart review | CPM |
| Assessment of health-related quality of life | Process | Chart review | CPM |
| Number of patients developing blood leak during dialysis treatment | Surrogate outcome | Chart review | 1 |
| Number of prescriptions of dialysate fluid according to lab values | Process | Chart review | 1 |
| Completed number of HCFA Death Notification forms with all specified mandatory items | Process | Chart review | 2 |
| Completed number of HCFA Medical Evidence Report forms with all specified mandatory items | Process | Chart review | 2 |
| Number of HCFA Death Notification forms within 30 days of the patient’s death date | Process | Chart review | 2 |
| Number of HCFA Medical Evidence Report forms within 45 days of dialysis start date | Process | Chart review | 2 |
| Patient vascular access arm correctly washed | Process | Chart review | 35 |
| Review of possible complications in the interdialytic period | Process | Chart review | 35 |
| Check the needle size is as prescribed | Process | Chart review | 35 |
| Search for signs of infection in case with catheter use | Process | Chart review | 35 |
| Vascular access assessment for patients with arteriovenous fistula or graft | Process | Chart review | 35 |
| Check that blood pressure and heart rate measurements were recorded as planned | Process | Chart review | 35 |
| Check that the prescribed dry weight was reached | Process | Chart review | 35 |
| Absence of residual disinfection agent | Process | Chart review | 35 |
| Check for the absence of compliance related to possible errors | Process | Chart review | 35 |
| Check that all dialysis parameters are entered as prescribed | Process | Chart review | 35 |
| Check that dialyzer type is as prescribed | Process | Chart review | 35 |
| Check that the patient did not receive unnecessary punctures | Process | Chart review | 35 |
| Check that the prescribed treatment time was delivered with a maximum tolerance of 10 min | Process | Chart review | 35 |
| Initiation of treatment within 15 min from the scheduled time | Process | Chart review | 35 |
| Chart audit | Process | Chart review | 40 |

Note: The reference article numbers refer to the list of included articles shown in Supplementary Text 2. The indicators referenced as CPM include those related to Health Care Finance Administration or Centers for Medicare and Medicaid Services.

Abbreviations: Hb, hemoglobin; Ht, hematocrit; CPM, clinical performance measure; ESA, erythropoietin stimulating agent; TSAT, transferrin saturation; Ca, calcium; P, phosphorus; PTH, parathyroid hormone; ALP, alkaline phosphatase; URR, urea reduction ratio; AVF, arteriovenous fistula; HbA1c, hemoglobin A1c; CRP, C-reactive protein; CHAPS, consumer assessment of healthcare providers and systems; HCFA, health care finance administration
