# Supplementary material for: Variations and characteristics of quality indicators for maintenance hemodialysis patients: A systematic review
Source: Health Sci Rep. 2018 Sep 5;1(11):e89. doi: 10.1002/hsr2.89 (PMC6242363; doi:10.1002/hsr2.89)
Supplement: Supplementary file 2 — Text S1. Search strategies for electronic literature search [file HSR2-1-e89-s002.docx]

**Supplementary Text 1. Search strategies for electronic literature search**

a) MEDLINE via Pubmed/CENTRAL

#1 quality indicator, health care [Mesh Terms]

#2 “clinical indicator”

#3 “clinical indicators”

#4 “process indicator”

#5 “process indicators”

#6 “performance indicator”

#7 “performance indicators”

#8 #1 or #2 or #3 or #4 or #5 or #6 or #7

#9 renal insufficiency, chronic [Mesh Terms]

#10 renal replacement therapy

#11 dialysis

#12 hemodialysis

#13 haemodialysis

#14 kidney failure

#15 kidney disease

#16 renal failure

#17 renal disease

#18 #9 or #10 or #11 or #12 or #13 or #14 or #15 or #16 or #17

#19 #8 and #18

b) SCOPUS

#1 “quality indicator”

#2 “quality indicators”

#3 “clinical indicator”

#4 “clinical indicators”

#5 “performance indicator”

#6 “performance indicators”

#7 #1 or #2 or #3 or #4 or #5 or #6

#8 renal insufficiency

#9 renal replacement therapy

#10 dialysis

#11 hemodialysis

#12 haemodialysis

#13 kidney failure

#14 kidney disease

#15 renal failure

#16 renal disease

#17 #8 or #9 or #10 or #11 or #12 or #13 or #14 or #15 or #16

#18 #7 and #17

c) CINAHL

#1 Clinical Indicators [MH]

#2 “quality indicator”

#3 “quality indicators”

#4 “clinical indicator”

#5 “clinical indicators”

#6 “process indicator”

#7 “process indicators”

#8 “performance indicator”

#9 “performance indicators”

#10 #1 or #2 or #3 or #4 or #5 or #6 or #7 or #8 or #9

#11 Renal Insufficiency, Chronic+ [MH]

#12 renal replacement therapy

#13 dialysis

#14 hemodialysis

#15 haemodialysis

#16 kidney failure

#17 kidney disease

#18 renal failure

#19 renal disease

#20 #11 or #12 or #13 or #14 or #15 or #16 or #17 or #18 or #19

#21 #10 and #20
