# Supplementary material for: Variations and characteristics of quality indicators for maintenance hemodialysis patients: A systematic review
Source: Health Sci Rep. 2018 Sep 5;1(11):e89. doi: 10.1002/hsr2.89 (PMC6242363; doi:10.1002/hsr2.89)
Supplement: Supplementary file 3 — Text S2. References of the included articles [file HSR2-1-e89-s003.docx]

**Supplementary Text 2. References of the included articles**

1. Patton S, Stanley J. Bridging quality assurance and continuous quality improvement. J Nurs Care Qual. 1993 Jan;7(2):15–23.

2. Armistead N, Mays C. 1999 facility Profile Reports. Adv Ren Replace Ther. 2000 Oct;7(4 Suppl 1):S31-35.

3. Bogdanski P, Locking-Cusolito H. Continuous quality improvement: at the grassroots. CANNT J. 2001 Dec;11(4):44–7.

4. Bonucchi D, Ferramosca E, Ciuffreda A, et al. Evaluation of dialysis access care by means of process quality indicators. J Vasc Access. 2000 Mar;1(1):6–9.

5. Capelli JP. Implementing organizational systems to measure outcome-related processes of end-stage renal disease care. Am J Kidney Dis. 1994 Aug;24(2):346–54.

6. Coelho AP, Sá HO, Diniz JA, Dussault G. The integrated management for renal replacement therapy in Portugal. Hemodial Int. 2014 Jan;18(1):175–84.

7. Cormier T, Magat O, Hager S, Ng F, Lee M. Physiologic and psychosocial approaches to global management of the hemodialysis patient in the Southern Alberta Renal Program. CANNT J. 2012 Jun;22(2):36–41.

8. Diamant MJ, Young A, Gallo K, et al. Hemodialysis in a satellite unit: clinical performance target attainment and health-related quality of life. Clin J Am Soc Nephrol. 2011 Jul;6(7):1692–9.

9. Grangé S, Hanoy M, Le Roy F, Guerrot D, Godin M. Monitoring of hemodialysis quality-of-care indicators: why is it important? BMC Nephrol. 2013 May;14:109.

10. Hirth RA, Turenne MN, Wheeler JRC, Pan Q, Ma Y, Messana JM. Provider monitoring and pay-for-performance when multiple providers affect outcomes: An application to renal dialysis. Health Serv Res. 2009 Oct;44(5 Pt 1):1585–602.

11. Kõlvald K, Pechter U, Luman M, Ilmoja M, Ots-Rosenberg M. Improvements in renal replacement therapy practice patterns in estonia. Nephron Extra. 2014 Jan;4(2):108–18.

12. Lacson E, Xu J, Lin S-F, Dean SG, Lazarus JM, Hakim R. Association between achievement of hemodialysis quality-of-care indicators and quality-of-life scores. Am J Kidney Dis. 2009 Dec;54(6):1098–107.

13. Lowrie EG. Illustrating use of a clinical data system: the NMC-FMC system. Clin J Am Soc Nephrol. 2009 Dec;4 Suppl 1:S41-48.

14. Lynch SK, Ahanchi SS, Dexter DJ, Glickman MH, Panneton JM. Patient compliance limits the efforts of quality improvement initiatives on arteriovenous fistula maturation. J Vasc Surg. 2015 Jan;61(1):184–91.

15. Morsch CM, Gonçalves LF, Barros E. Health-related quality of life among haemodialysis patients--relationship with clinical indicators, morbidity and mortality. J Clin Nurs. 2006 Apr;15(4):498–504.

16. Mozes B, Shabtai E, Zucker D. Variation in mortality among seven hemodialysis centers as a quality indicator. Clin Perform Qual Health Care. 1998 Jun;6(2):73–8.

17. Parra E, Ramos R, Betriu A, Paniagua J, Belart M, Martínez T. Effect of a quality improvement strategy on several haemodialysis outcomes. Nephrol Dial Transplant. 2008 Sep;23(9):2943–7.

18. Peter J, Finkelstein F. The effectiveness of low-dose maintenance i.v. iron therapy: a dialysis facility’s experience. Nephrol Nurs J. 2006 Feb;33(1):71–4, 90.

19. Plantinga LC, Fink NE, Jaar BG, et al. Attainment of clinical performance targets and improvement in clinical outcomes and resource use in hemodialysis care: a prospective cohort study. BMC Health Serv Res. 2007 Jan;7:5.

20. Plantinga LC, Jaar BG, Fink NE, et al. Frequency of patient-physician contact in chronic kidney disease care and achievement of clinical performance targets. Int J Qual Health Care. 2005 Apr;17(2):115–21.

21. Richards N, Ayala JA, Cesare S, et al. Assessment of quality guidelines implementation using a continuous quality improvement programme. Blood Purif. 2007;25(3):221–8.

22. Saudan P, Kossovsky M, Halabi G, Martin PY, Perneger TV, Western Switzerland Dialysis Study Group. Quality of care and survival of haemodialysed patients in western Switzerland. Nephrol Dial Transplant. 2008 Jun;23(6):1975–81.

23. Tan J. Renal replacement therapy in Brunei Darussalam: comparing standards with international renal registries. Nephrol (Carlton). 2014 May;19(5):288–95.

24. Thompson S, Bello A, Wiebe N, et al. Quality-of-care indicators among remote-dwelling hemodialysis patients: a cohort study. Am J Kidney Dis. 2013 Aug;62(2):295–303.

25. Wazny LD, Raymond CB, Lesperance EM, Bernstein KN. Are CSN and NKF-K/DOQI mineral metabolism guidelines for hemodialysis patients achievable? Results from a provincial renal program. CANNT J. 2008 Jun;18(2):36–41, 44-50; quiz 42-43, 51–2.

26. Wilson SM, Robertson JA, Chen G, et al. The IMPACT (Incident Management of Patients, Actions Centered on Treatment) program: a quality improvement approach for caring for patients initiating long-term hemodialysis. Am J Kidney Dis. 2012 Sep;60(3):435–43.

27. Wintz R, Rosenthal B, Fadem SZ. The Physician Quality Reporting Initiative: a practical approach to implementing quality reporting. Adv Chronic Kidney Dis. 2008 Jan;15(1):56–63.

28. Benner D, Hollister D, Mcallister CJ, Thiry K. The DaVita Quality Index (DQI): a measure of clinical performance. Dial Transplant. 2003;32(5):269–273.

29. Couchoud C, Kooman J, Finne P, et al. From registry data collection to international comparisons: examples of haemodialysis duration and frequency. Nephrol Dial Transplant. 2009 Jan;24(1):217–24.

30. Di Benedetto A, Richards N, Marcelli D, et al. Is it necessary to check outcomes to improve quality of care? The example of anemia management. J Nephrol. 2008 Apr;21 Suppl 13:S146-152.

31. Hoar S, Morton AR, Meers C, Mulkerns S, Lawlor M, Toffelmire EB. A multidisciplinary approach to the control of the calcium X phosphate product in dialysis patients. Dial Transplant. 1999;28(6):309–313.

32. Ilumin MP. A continuous quality improvement (CQI) initiative: focusing on primary nurse accountability. Nephrol Nurs J. 2003 Feb;30(1):33–7.

33. Lindberg M, Prütz K-G, Lindberg P, Wikström B. Interdialytic weight gain and ultrafiltration rate in hemodialysis: lessons about fluid adherence from a national registry of clinical practice. Hemodial Int. 2009 Apr;13(2):181–8.

34. Ludvigsen MS, Hermansen HM, Lindberg M. The quality of nursing care during intradialytic fluid removal in haemodialysis: time to change practice? J Clin Nurs. 2015 Jun;24(11–12):1733–6.

35. Marcelli D, Matos A, Sousa F, et al. Implementation of a quality and safety checklist for haemodialysis sessions. Clin Kidney J. 2015 Jun;8(3):265–70.

36. Ponce P, Marcelli D, Guerreiro A, et al. Converting to a capitation system for dialysis payment--the Portuguese experience. Blood Purif. 2012;34(3–4):313–24.

37. Saudan P, Halabi G, Perneger T, et al. Variability in quality of care among dialysis units in western Switzerland. Nephrol Dial Transplant. 2005 Sep;20(9):1854–63.

38. van Andringa de Kempenaer T, ten Have P, Oskam J. Improving quality of vascular access care for hemodialysis patients. Jt Comm J Qual Saf. 2003 Apr;29(4):191–8.

39. Waeleghem JP, Elseviers MM, Vos JY, Malderen P. Quality assessment in nephrology nursing. J Ren Care. 2000;26(4):46–51.

40. Yuan CM, Prince LK, Zwettler AJ, Nee R, Oliver JD, Abbott KC. Assessing achievement in nephrology training: using clinic chart audits to quantitatively screen competency. Am J Kidney Dis. 2014 Nov;64(5):737–43.

41. 2000 Annual Report: ESRD Clinical Performance Measures Project. Am J Kidney Dis. 2001 Apr;37(4):S1.

42. Hemodialysis CPMs IV and V: results from the pilot-test of the Facility Questionnaire, 1999-2000: supplemental report #2: 2000 ESRC Clinical Performance Measures Project. Nephrol Nurs J. 2001 Aug;28(4):471–3.

43. Executive summary: ESRD Clinical Performance Measures Project 2001 annual report. Nephrol Nurs J. 2002 Feb;29(1):45–8.

44. Sehgal AR. Impact of quality improvement efforts on race and sex disparities in hemodialysis. JAMA. 2003 Feb;289(8):996–1000.

45. Kelly MP. Use of dietetic-specific nutritional diagnostic codes in clinical reasoning relevant to the nutritional management of core clinical outcome indicators in hemodialysis patients. Adv Chronic Kidney Dis. 1997;4(2):125–135.

46. Rocco MV, Frankenfield DL, Hopson SD, McClellan WM. Relationship between clinical performance measures and outcomes among patients receiving long-term hemodialysis. Ann Intern Med. 2006 Oct;145(7):512–9.

47. Coladonato JA, Frankenfield DL, Reddan DN, et al. Trends in anemia management among US hemodialysis patients. J Am Soc Nephrol. 2002 May;13(5):1288–95.

48. Fishbane S, Miller I, Wagner JD, Masani NN. Changes to the end-stage renal disease quality incentive program. Kidney Int. 2012 Jun;81(12):1167–71.

49. Frankenfield D, Johnson CA, Wish JB, Rocco MV, Madore F, Owen WF. Anemia management of adult hemodialysis patients in the US results: from the 1997 ESRD Core Indicators Project. Kidney Int. 2000 Feb;57(2):578–89.

50. Frederick PR, Frankenfield DL, Biddle MG, Sims TW. Changes in dialysis units’ quality improvement practices from 1994 to 1996. ANNA J. 1998 Oct;25(5):469–78.

51. Frederick PR, Maxey NL, Clauser SB, Sugarman JR. Developing dialysis facility-specific performance measures for public reporting. Health Care Financ Rev. 2002;23(4):37–50.

52. Krishnan M, Brunelli SM, Maddux FW, et al. Guiding principles and checklist for population-based quality metrics. Clin J Am Soc Nephrol. 2014 Jun;9(6):1124–31.

53. Lowrie EG, Teng M, Lacson E, Lew N, Lazarus JM, Owen WF. Association between prevalent care process measures and facility-specific mortality rates. Kidney Int. 2001 Nov;60(5):1917–29.

54. McClellan WM, Frankenfield DL, Frederick PR, et al. Can dialysis therapy be improved? A report from the ESRD Core Indicators Project. Am J Kidney Dis. 1999 Dec;34(6):1075–82.

55. McClellan WM, Frankenfield DL, Frederick PR, Helgerson SD, Wish JB, Sugarman JR. Improving the care of ESRD patients: a success story. Health Care Financ Rev. 2003;24(4):89–100.

56. McClellan WM, Soucie JM, Krisher J, Caruana R, Haley W, Farmer C. Improving the care of patients treated with hemodialysis: a report from the Health Care Financing Administration’s ESRD Core Indicators Project. Am J Kidney Dis. 1998 Apr;31(4):584–92.

57. Owen WF, Szczech L, Johnson C, Frankenfield D. National perspective on iron therapy as a clinical performance measure for maintenance hemodialysis patients. Am J Kidney Dis. 1999 Oct;34(4 Suppl 2):S5–11.

58. Saunders MR, Chin MH. Variation in dialysis quality measures by facility, neighborhood, and region. Med Care. 2013 May;51(5):413–7.

59. Stoffel MP, Barth C, Lauterbach KW, Baldamus CA. Evidence-based medical quality management in dialysis--Part I: Routine implementation of QiN, a German quality management system. Clin Nephrol. 2004 Sep;62(3):208–18.

60. Tangri N, Moorthi R, Tighiouhart H, Meyer KB, Miskulin DC. Variation in fistula use across dialysis facilities: is it explained by case-mix? Clin J Am Soc Nephrol. 2010 Feb;5(2):307–13.

61. Van Wyck D, Robertson J, Nissenson A, Provenzano R, Kogod D. Relationship among length of facility ownership, clinical performance, and mortality. Clin J Am Soc Nephrol. 2010 Feb;5(2):248–51.

62. Clinical indicators & preventive health. Am J Kidney Dis. 2011;57(1 SUPPL. 1):e287–300.

63. Clinical Indicators and Preventive Care. Am J Kidney Dis. 2012 Jan 1;59(1):e195–204.

64. Clinical Indicators & Preventive Care. Am J Kidney Dis. 2013 Jan 1;61(1):e229–36.

65. Clinical Indicators & Preventive Care. Am J Kidney Dis. 2014 Jan 1;63(1):e229–36.

66. Chapter 3: Clinical Indicators and Preventive Care. Am J Kidney Dis. 2015 Jul 1;66(1):S129–38.

67. Fishbane S, Hazzan A. Meeting the 2012 QIP (Quality Incentive Program) clinical measures: strategies for dialysis centers. Am J Kidney Dis. 2012 Nov;60(5 Suppl 1):S5-13; quiz S14-17.

68. McClellan WM, Frederick PR, Helgerson SD, Hayes RP, Ballard DJ, McMullan M. A data-driven approach to improving the care of in-center hemodialysis patients. Health Care Financ Rev. 1995;16(4):129–40.

69. Spolter YS, Seliger SL, Zhan M, Hsu VD, Walker LD, Fink JC. The relationship between dialysis performance measures: adequacy and anemia management. Am J Kidney Dis. 2007 Nov;50(5):774–81.

70. Stevens G, Meier BJ, McCarthy JT, Carlson D. Using comparative facility review to improve dialysis patient outcomes. Dial Transplant. 1996;25(9):568–578.

Note: Articles related to Health Care Finance Administration or Centers for Medicare & Medicaid Services include (41), (42), (43), (44), (45), (46), (47), (48), (49), (50), (51), (52), (53), (54), (55), (56), (57), (58), (59), (60), (61), (62), (63), (64), (65), (66), (67), (68), (69) and (70).
